# From canopy to seed: Loss of snow drives directional changes in forest composition

**DOI:** 10.1002/ece3.5383

**Published:** 2019-06-19

**Authors:** Sarah M. Bisbing, Brian J. Buma, Lauren E. Oakes, John Krapek, Allison L. Bidlack

**Affiliations:** ^1^ Department of Natural Resources and Environmental Science University of Nevada – Reno Reno Nevada USA; ^2^ Department of Integrative Biology University of Colorado, Denver Denver Colorado USA; ^3^ Department of Earth System Science Stanford University Stanford California USA; ^4^ Climate Change Americas Program Wildlife Conservation Society Bozeman Montana USA; ^5^ Department of Natural Sciences University of Alaska Southeast Juneau Alaska USA; ^6^ Alaska Coastal Rainforest Center University of Alaska Southeast Juneau Alaska USA

**Keywords:** Callitropsis nootkatensis, climate change, community composition, diversity, forest mortality, yellow‐cedar decline

## Abstract

Climate change is altering the conditions for tree recruitment, growth, and survival, and impacting forest community composition. Across southeast Alaska, USA, and British Columbia, Canada, *Callitropsis nootkatensis* (Alaska yellow‐cedar) is experiencing extensive climate change‐induced canopy mortality due to fine‐root death during soil freezing events following warmer winters and the loss of insulating snowpack. Here, we examine the effects of ongoing, climate‐driven canopy mortality on forest community composition and identify potential shifts in stand trajectories due to the loss of a single canopy species. We sampled canopy and regenerating forest communities across the extent of *C. nootkatensis* decline in southeast Alaska to quantify the effects of climate, community, and stand‐level drivers on *C. nootkatensis* canopy mortality and regeneration as well as postdecline regenerating community composition. Across the plot network,* C. nootkatensis* exhibited significantly higher mortality than co‐occurring conifers across all size classes and locations. Regenerating community composition was highly variable but closely related to the severity of *C. nootkatensis* mortality. *Callitropsis nootkatensis* canopy mortality was correlated with winter temperatures and precipitation as well as local soil drainage, with regenerating community composition and *C. nootkatensis* regeneration abundances best explained by available seed source. In areas of high *C. nootkatensis* mortality, *C. nootkatensis* regeneration was low and replaced by *Tsuga*. Our study suggests that climate‐induced forest mortality is driving alternate successional pathways in forests where *C. nootkatensis* was once a major component. These pathways are likely to lead to long‐term shifts in forest community composition and stand dynamics. Our analysis fills a critical knowledge gap on forest ecosystem response and rearrangement following the climate‐driven decline of a single species, providing new insight into stand dynamics in a changing climate. As tree species across the globe are increasingly stressed by climate change‐induced alteration of suitable habitat, identifying the autecological factors contributing to successful regeneration, or lack thereof, will provide key insight into forest resilience and persistence on the landscape.

## 1 INTRODUCTION

Climate change is altering the conditions for tree recruitment, growth, and survival, and range shifts are a widely anticipated consequence of novel temperature and precipitation regimes. Local adaptation to historical climate is already creating mismatches between species’ current distributions and suitable habitat conditions (Aitken & Bemmels, 2015; Aitken, Yeaman, Holliday, Wang, & Curtis‐McLane, 2008), and these disparities are likely to be most extreme for seedlings, which have a narrower range of tolerance to climate conditions than mature individuals (Niinemets, 2010). As climate regimes shift, habitat suitable for survival of mature trees and the conditions necessary for germination and establishment may no longer correspond with each other, potentially leading to simultaneous canopy mortality and declines in regeneration, and, ultimately, shifts in species distributions (Walck, Hidayati, Dixon, Thompson, & Poschlod, 2011). The key to regeneration success and long‐term survival will be continued synchronization of tree germination, establishment, and growth with local climate (Aitken & Bemmels, 2015). In contrast, asynchronization will likely lead to restructuring of forest communities through dieback (Oakes, Hennon, O'Hara, & Dirzo, 2014), regeneration failures (Holz, Wood, Veblen, & Bowman, 2015), or both (Anderegg, Kane, & Anderegg, 2013). Inhibition of conifer regeneration, for example, could lead to ecosystem type conversions (Allen & Breshears, 1998; Holz et al., 2015) and long‐term changes in stand dynamics (Turner, Dale, & Everham, 1997). Thus, understanding the relative climatic tolerances of the mature and regeneration life phases of species is critical to predicting their response to climate change.

While the driving processes are difficult to disentangle, species range contractions consist of concurrent or sequential canopy mortality and regeneration failures. Climate change‐induced canopy mortality is already a globally documented phenomenon (Allen, Breshears, & McDowell, 2015; Allen et al., 2010). Altered precipitation regimes (i.e., change in timing, amount, frequency, type) combined with simultaneous increases in temperature are leading to both drought mortality (Guarín & Taylor, 2005; Peng et al., 2011) and, at the other extreme, mortality attributed to earlier snowmelt and spring freezing events (Bourque, Cox, Allen, Arp, & Meng, 2005). Drought‐induced canopy mortality is well‐established (Allen et al., 2015; Anderegg et al., 2013), and recent studies have quantified the impacts of prolonged drought (Redmond, Weisberg, Cobb, & Clifford, 2018) and postfire drought conditions (Stevens‐Rumann et al., 2017; Young et al., 2019) on regeneration. However, relatively little is known about the consequences of reduced snowpack and early season frost damage on canopy mortality and regeneration response, although these phenomena may become more common as the climate warms (Woldendorp, Hill, Doran, & Ball, 2008)—leading to root mortality and nutrient loss (Decker, Wang, Waite, & Scherbatskoy, 2003), needle and bud injury (Man, Kayahara, Dang, & Rice, 2009), canopy mortality (Buma, 2018), and seedling mortality (Camarero & Gutiérrez, 2004). Regardless of the climatic stressor, widespread canopy mortality is likely to be ongoing under the more extreme conditions predicted for the future, and under such conditions, range contraction potential will be governed by regeneration success or failure.

Not all climate‐driven mortality will result in a range contraction, as regeneration after extensive mortality is dependent upon two conditions: (a) available sources of seed and/or vegetative reproduction and (b) the establishment environment (climatic, abiotic, and biotic). Seed supply generally decreases with a loss of mature trees (Tepley, Veblen, Perry, Stewart, & Naficy, 2016), and this loss can result in reductions in seedling abundances, lower recruitment into mature tree size classes, and the potential for local extirpation due to competition with nondecline‐affected species (Oakes et al., 2014). Mortality of mature trees may, conversely, create more favorable establishment conditions by increasing available light and releasing advanced regeneration (Macek et al., 2017; Zeppenfeld et al., 2015) or leading to more successful germination and subsequent survival (Whitmore, 1989). Such increases in favorable microsites may partially offset canopy declines; under such conditions, regeneration of species declining in the canopy may increase despite a reduction in available seed—similar to pulses of recruitment following windthrow events (Dunn, Guntenspergen, & Dorney, 1983; Peterson & Pickett, 1995). Comparing the relative strength of seed source versus community competition and abiotic changes is therefore important in predicting the net effects of mortality and the potential for range shifts at broader scales—thus, resilience.

The effects of canopy mortality on regeneration are, however, difficult to isolate, as climate change‐induced canopy mortality often leads to concurrent decline of multiple species. Concurrent declines challenge our ability to identify the factors driving reductions in suitable habitat for mature tree survival versus those leading to regeneration failures for individual species, as a variety of interspecific relationships are changing simultaneously. Simplified systems in which a single species undergoes climate‐related mortality in isolation of climate effects on co‐occurring species provide a means of parsing out the effects of climate change on mortality, regeneration, and the resultant ecological community.

Across the North Pacific coastal temperate rainforest (NPCTR) of southeast Alaska, USA, and British Columbia, Canada, *Callitropsis nootkatensis,* D. Don, Oesrt. Ex D.P. Little (Alaska yellow‐cedar) is experiencing extensive, climate change‐driven mortality over more than 400,000 ha and ten degrees of latitude (Buma et al., 2017). Regional wintertime temperatures average 0°C; thus, a slight warming results in significant snow loss (Buma, 2018). Subfreezing weather events following snowmelt in late winter and early spring kills fine roots of mature *C. nootkatensis* (Hennon et al., 2016; Schaberg, Hennon, D'amore, & Hawley, 2008), and this phenomenon has been ongoing for several decades (Beier, Sink, Hennon, D'Amore, & Juday, 2008; Hennon, D'Amore, Schaberg, Wittwer, & Shanley, 2012). Mortality drivers in this complex pathway include the limited cold tolerance of roots, a reduction in insulating snowpack due to warmer winters, soil freezing due to lack of insulation, and a positive feedback loop in which canopy gaps in declining forests lead to further reductions in springtime snowpack (Beier et al., 2008; Schaberg et al., 2008). Given predicted future climate conditions, *C. nootkatensis* is likely to experience continued decline over most of its range (Buma, 2018) and be replaced by other regionally dominant conifers (Oakes et al., 2014). Co‐occurring *Tsuga heterophylla* Raf. (Sarg) (western hemlock) and *Picea sitchensis* Bong. (Carr) (Sitka spruce) are not considered sensitive to snow loss (Buma & Barrett, 2015) and may increase in dominance when *C. nootkatensis* fails to regenerate (Oakes et al., 2014). Yet, despite extensive research on the drivers of decline (Barrett, Latta, Hennon, & Eskelson, 2012; Buma et al., 2017; Hennon et al., 2012; Hennon, Hansen, & Shaw, 1990; Hennon & Shaw, 1997; Hennon, Shaw, & Hansen, 1990; Schaberg et al., 2008), little is known about *C. nootkatensis* regeneration following canopy mortality, and the fate of *C. nootkatensis* and long‐term dynamics of affected forests remain unknown.

To address these knowledge gaps and isolate the conditions differentiating habitat suitable for mature tree survival versus those key to successful regeneration in a climate mortality‐affected system, we sampled declining *C. nootkatensis* forests over a five‐degree latitude range in the NPCTR of southeast Alaska to ask the following: (a) “Does climate‐induced mortality occur across all size classes of the affected species, and, specifically, does regeneration response correspond with the same climate conditions driving mortality?”, (b) “If response is differential, what climatic and community factors drive tree mortality versus postdecline composition of the regenerating community?”, and (c) “Is community composition stable or in the process of a decline‐induced shift?”. Our analysis provides new insight into stand dynamics in a changing climate by increasing understanding of forest ecosystem response and rearrangement following the decline of a single species. Obtaining information on life stage response to climate‐induced mortality, postdecline community composition, and stand dynamics in the NPCTR and beyond will be essential to scientifically based forest management and vital to supporting conservation efforts in the face of climate change.

## 2 METHODS

### 2.1 Study area and species

*Callitropsis nootkatensis* is distributed across ~20° of latitude from northern California into Prince William Sound, Alaska (DellaSala et al., 2011). Half of the species’ range occurs in the perhumid region of the NPCTR (10° of the 20° latitudinal distribution), where this study occurs, which is characterized by mild, consistently humid conditions and high annual precipitation (3,182 mm average, 621–9,332 range; extracted from ClimateWNA, Wang, Hamann, Spittlehouse, & Carroll, 2016). Across the NPCTR, *C. nootkatensis* co‐occurs with *Picea sitchensis*, *Pinus contorta ssp. contorta* Douglas Ex. Louden (shore pine), *Thuja plicata* Donn ex D. Don (western redcedar), *Tsuga heterophylla,* and *Tsuga mertensiana* (Bong.) Carriere. *Sphagnum* spp. are common in areas of poor drainage and low forest productivity, decreasing in abundance with increasing slope and increasing depth to groundwater (Bisbing, Cooper, D'Amore, & Marshall, 2016; Neiland, 1971).

*Callitropsis nootkatensis* is locally distributed across the NPCTR's hydrologic gradient from emergent wetlands to upland forests, which corresponds to a gradient of low to high forest productivity (Hennon et al., 2012, 2016). This gradient drives the distribution, abundance, and biomass of the region's dominant tree species (Bisbing et al., 2016), with low‐lying saturated peatlands limiting the success of most species but providing low‐competition environments for stress‐tolerant species, such as *P. contorta* (Bisbing et al., 2016) and *C. nootkatensis* (Caouette et al., 2015; Hennon, Hansen, et al., 1990). Background mortality rates in healthy *C. nootkatensis* forests average <25% (Hennon, Hansen, et al., 1990).

*Callitropsis nootkatensis* regeneration occurs via seed but also through vegetative reproduction. Vegetative layering is particularly common on lower‐productivity peatlands with poor drainage and/or high snow cover; lower limbs will produce adventitious roots when depressed by accumulating *Sphagnum* and snow (Hennon et al., 2016). Individuals recruited through vegetative layering often persist on these lower‐productivity peatlands despite mature tree mortality.

### 2.2 Plot design and sampling

**Figure 1:**
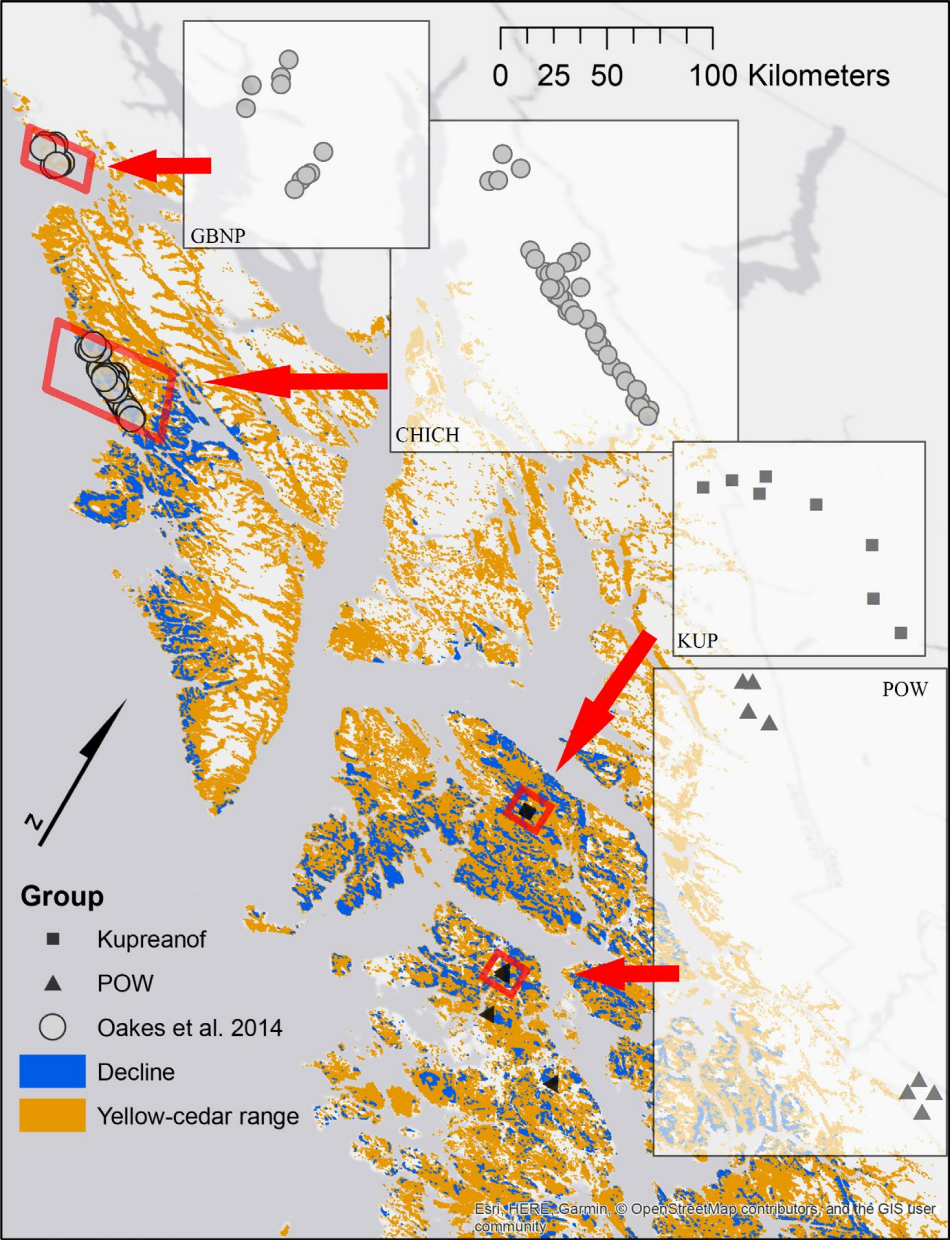
Map of *Callitropsis nootkatensis* plot network distributed across southeast Alaska. Previously established plots were installed by Oakes and colleagues in 2011 and 2012 in Glacier Bay National Park (GBNP) and Chichagof Island (CHICH), and additional plots were installed by the authors in 2015 and 2016 on Kupreanof Island (KUP) and Prince of Wales Island (POW). Inset plots for illustration of the numerical distribution of plots only (scale varies). For a complete range and decline map, see Buma et al. (2017) or Buma (2018)

A total of 67 plots were compiled from published (Oakes et al., 2014) and ongoing research by the authors in the perhumid NPCTR subregion of southeast Alaska (Figure 1). This plot network represents all compatible studies in the region, with compatible defined as those including both regeneration and canopy community data, precise locations, comparable methodologies, and comparable scales. Due to aggregating across sources and studies, each with their own goals, plots were not randomly distributed across the region. However, the data products were compatible and collectively allowed for analysis of regeneration response to the canopy decline severity gradient.

Plots installed by Oakes and colleagues (2014, *n* = 50) were randomly selected from areas stratified by *C. nootkatensis* status—live forests (*n* = 20) and three time‐since‐mortality classes (*n* = 10 each in recent, mid‐range, and old) on Chichagof Island and in Glacier Bay National Park (installed in 2011–2012, Figure 1). These plots represent the northernmost extent of the contiguous species distribution while also approaching the northernmost extent of documented decline. Data were collected in nested, fixed‐radius plots: (a) 10.3m radius (~333 m^2^) for mature trees (>25 cm diameter at breast height, DBH) and (b) 6m radius (~113 m^2^) for saplings (<2.5 cm DBH and >1 m in height), treelets (2.5–9.9 cm DBH), and small trees (10–24.9 cm DBH, see Oakes et al., 2014 for details). In addition, conifer germinants and seedlings (<10 cm height) were sampled using eight one‐m^2^ quadrats (8m^2^ total area sampled) installed in the cardinal directions at five and eight meters from plot center.

To extend inference across *C. nootkatensis*' distribution in southeast Alaska, we leveraged 17 additional nested, fixed‐radius plots on Kupreanof and Prince of Wales Islands (installed 2015–2016, Figure 1). Mature and small trees (>10 cm DBH) were sampled over a 400‐m^2^ plot. Treelets, saplings, and advanced regeneration (>10 cm but <1.37m in height) were sampled in a nested 100‐m^2^ plot. Germinants and seedlings (<10 cm height) were tallied by species in four one‐m^2^ quadrats at the corners of the 400‐m^2^ plot (16 m^2^ total area sampled). Hereafter, the term regeneration refers to the combined germinant and seedling classes, including all recently emerged germinants (i.e., cotyledons still visible) to seedlings less than 10cm in height and, presumably, <2 years of age.

Across all plots, species, DBH, tree status (live/dead), live crown condition, and snag class were sampled for all trees. Snags were identified to species, if possible, and *C. nootkatensis* snags classified into time‐since‐mortality classes according to Hennon, Shaw, et al. (1990). Unidentifiable snags were classified as unknown species. Additionally, regeneration was identified to the genus for *Tsuga* germinants (*heterophylla* and *mertensiana*) but to the species for all other species and size classes. Disturbed areas (e.g., windthrow, landslides) were eliminated from plot selection to avoid the confounding influence of disturbance on community composition.

### 2.3 Environmental variables

Climate data were extracted from ClimateWNA v5.51 (http://www.climatewna.com, Wang et al., 2016), which provides spatially interpolated, locally downscaled climate data and derived biologically relevant climate variables. Data were obtained for three date ranges to capture drivers of ongoing decline and regeneration—the last few decades (30‐year normal, 1981–2010), the five‐year period prior to and including sampling year (five‐year average, range 2007–2016), and the year of sampling (annual, range: 2011–2016). Annual data were used to evaluate the influence of current year conditions on the regenerating community, while data from the five‐year period were used to quantify the influence of multiyear climate on canopy mortality, potential seed availability and viability, and regenerating community composition. A five‐year window was selected for two reasons: (a) *C. nootkatensis* cones take two to three years to mature and produce viable seed (Bonner & Karrfalt, 2008), and (b) we focus regeneration analysis on individuals presumed to be up to two years of age.

Prior to analysis, we ran a correlation analysis to reduce the full set of ClimateWNA variables to a minimally correlated set (<0.65) while retaining those considered biologically important in *C. nootkatensis* decline, including winter and spring temperatures and precipitation, length of the growing season, and frost period (Buma et al., 2017; Hennon et al., 2012). The following variables were selected as potential predictors: winter (December–February) and spring (March–May) minimum and maximum temperatures (°C); winter, spring, summer (June–August), and average annual precipitation (mm); number of frost‐free days in winter and spring (days); average annual length of growing season (frost‐free days), date of first frost (Julian date); and annual precipitation as snow (mm; Table 1).

**Table 1:** Potential predictor variables included in regression tree modeling of *Callitropsis nootkatensis* (CANO) mortality and regeneration abundance following decline

Factors	Variables	Unit
Location	Group	Study area
Time	Time since onset of mortality	Years
Competition	CANO, PICO, PISI, THPL, TSHE, TSME	Live basal area in m^2^/ha
	CANO, PICO, PISI, THPL, TSHE, TSME, Unknown, and Unknown *Tsuga*	Dead basal area in m^2^/ha
	Total live basal area	m^2^/ha
	Total dead basal area	m^2^/ha
	*Sphagnum* coverage	Percent
Climate (annual, 5‐year period, 30‐year normal from 1981 to 2010)	Maximum and minimum temperatures: winter, spring	C
	Precipitation: winter, spring, summer, annual	mm
	Length of growing season	Days
	Frost‐free days: winter, spring	Days
	Date of first frost	Julian Date
	Precipitation as snow	mm
Topographic	Elevation	Meters
	Aspect	Degrees
	Slope	Degrees
	Contributing area	Log_10_ (m^2^)
	Wind exposure (Buma & Barrett, 2015)	1–8
	Landslide likelihood (Buma & Johnson, 2015)	0–1

Abbreviations: PICO, *Pinus contorta* spp. *contorta*; PISI, *Picea sitchensis*; THPL, *Thuja plicata*; TSHE, *Tsuga heterophylla*; and TSME, *Tsuga mertensiana*.

Local topographic data were obtained from the NASA ASTER mission (30m resolution, LP DAAC 2017) and used to derive elevation, slope, and aspect. To assess the role of local soil drainage, which influences competition and rooting, we selected two metrics operating at different scales. At the local scale, we used *Sphagnum* coverage. To do so, we sampled bryophyte (*n* = 67) and *Sphagnum* (subset, *n* = 12) cover on replicate 1‐m^2^ quadrats (8–10 per plot). Bryophyte coverage was correlated with *Sphagnum*‐specific coverage, so, on plots with bryophyte coverage only, a log‐linear model was created to estimate *Sphagnum* coverage (*R* ^2^ = 0.48, *F* = 15.81 on 1,15 *df*, *p* < 0.001). Predicted *Sphagnum* coverages were used in overall model creation. At the landscape scale, we used contributing area derived from the ASTER elevation data, a metric of upslope area potentially contributing runoff to a location in which higher values indicate wetter, lower‐lying areas. All data processing and subsequent analyses were conducted in R version 3.4.1. (R Core Team, 2018)

### 2.4 Data analyses

To control for the possibility of general forest declines, which would affect all species and obscure *C. nootkatensis*‐specific mortality, we used simple linear regressions to assess the correlation between the proportion of dead *C. nootkatensis* (log‐transformed) as compared to that of the other dominant co‐occurring tree species, and Kruskal–Wallis tests for nonparametric, ranked data to assess variation in proportional mortality among species, locations, and size classes (as defined above). There were no significant concurrent declines in any co‐occurring species (*p* > 0.05), so we focused subsequent mortality analyses on *C. nootkatensis* alone. We also used a Kruskal–Wallis analysis to test for significant differences in live tree abundances among species and locations. We then tested for significant differences in canopy and regenerating community composition among locations and as related to *C. nootkatensis* mortality with multivariate analysis of variance (MANOVA) tests using Bray–Curtis distances in the *vegan* package. The proportion of dead *C. nootkatensis* in the canopy (dead *C. nootkatensis* out of total *C. nootkatensis*) was also compared to regeneration density using a negative log‐transformed linear regression. In Kruskal–Wallis and MANOVA tests, proportional mortality was categorized into the following mortality severity classes: low = 1%–25%, moderate = 25.1%–69.9%, high = 70%–99%, and all = 100%.

Next, we compared drivers of *C. nootkatensis* canopy mortality versus regeneration. Two random forests analyses were run—one to model the proportion of dead *C. nootkatensis* in the canopy and another the abundance of *C. nootkatensis* regeneration. Both were based on potential drivers related to climate, disturbance exposure, topography, drainage, and competition (Table 1). Random forests, an extension of regression tree analysis (Breiman, 2001), are well suited for complex, nonlinear interactions between variables and generally perform better than other methods in predictive accuracy (Prasad, Iverson, & Liaw, 2006).

We took a two‐step process similar to importance‐based variable selection procedures (Evans & Cushman, 2009). First, an initial forest was grown using all potential variables, and variable importance was calculated, based on the decrease in accuracy on the out‐of‐bag sample (independent data points not used in building the tree used for testing) when each variable is permuted compared to the original tree. This is averaged across all trees in the forest. Variables were then assessed for cross correlation with each other. The top ten important uncorrelated variables (<0.65) were retained. This was necessary as, while random forests are not generally subject to overfitting due to correlated variables, correlation between variables often means several highly correlated variables may all be simultaneously considered of high importance. While this may not be an impediment to modeling accuracy (Fox et al., 2017), it interferes with our ability to interpret the random forest outputs versus our hypotheses. Instead, the top uncorrelated variables were used to create a second, final model. Our model was then investigated for the marginal influence of the most important variables on proportion dead and regeneration density by running the final model while varying the single variable of interest and plotting projected values (sometimes called a partial plot). We used the *randomForest* package for analyses.

To determine regenerating community types and identify potential shifts in community composition, we analyzed conifer regeneration abundances across all sampling locations using a cluster analysis with the Bray–Curtis distance measure and Ward's hierarchical agglomerative method in the *vegan* package. Community types were determined with an indicator species analysis within the *indicspecies* package; the appropriate number of community types was classified by maximizing the number of statistically significant indicator species in each group (Dufrêne & Legendre, 1997). We assessed differences in community composition and cluster types with Kruskal–Wallis tests for nonparametric, ranked data, including location, time since mortality, and proportional mortality severity class as potential predictors.

We then used a suite of nonparametric, multivariate analyses to compare patterns in and identify drivers of regenerating community composition. First, we performed nonmetric multidimensional scaling (NMS) ordinations on regeneration abundances based on Bray–Curtis dissimilarity with the *vegan* package (Oksanen et al., 2007). Nonmetric multidimensional scaling avoids the assumption of linear or unimodal responses so is well suited to non‐normal plant community data (McCune, Grace, & Urban, 2002). We employed permutational vector fitting (999 permutations) on biologically significant yet minimally correlated variables (<0.65, detailed above, Table 1) using a multiple linear regression technique with the *envfit* function to assess relationships between NMS ordinations of community structure and this reduced set of climate, community, and stand‐level variables. Variables identified as significant using *envfit* were then evaluated with a generalized additive model to test for linear fit, and variables representing nonlinear relationships were removed from the final model.

## 3 RESULTS

### 3.1 Community composition, canopy mortality, and regeneration

Tree diversity is generally low in the region (Caouette et al., 2015; Neiland, 1971), and despite the latitudinal range, all plots were similar in species composition. The canopy was a mixed‐conifer forest of *Tsuga (heterophylla* and/or *mertensiana*), *P. sitchensis,* and *C. nootkatensis*. *Picea sitchensis,* although common in the region, was rare on the plot network, with only one tree documented on many of the plots. *Pinus contorta* occurred in peatlands on Chichagof Island, while *T. plicata* was found only at higher elevations on Prince of Wales Island (other plots fell outside *T. plicata*'s range). Total basal area ranged from 9 to 87 m^2^/ha, and higher *C. nootkatensis* basal areas were generally found on higher productivity, upland forests or in areas north of the decline (Table A1). *Callitropsis nootkatensis* regeneration abundance ranged from 0 to 9.6 per m^2^ (mean 1.9/m^2^, median = 1/m^2^; Table A1).

**Figure 2:**
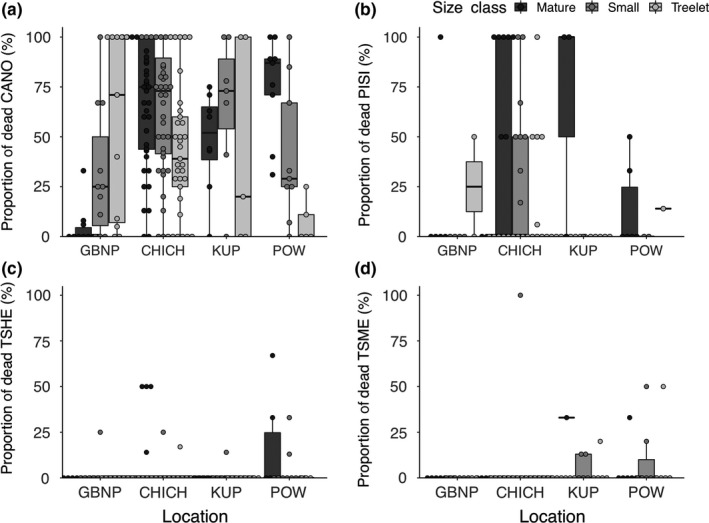
Proportional mortality (dots = plot‐level data, boxplot = median and range) of common canopy species of southeast Alaska: (a) *Callitropsis nootkatensis* (CANO), (b) *Picea sitchensis* (PISI), (c) *Tsuga heterophylla* (TSHE), and (d) *Tsuga mertensiana* (TSME). Proportional mortality was significantly different among species (*p* < 0.001) but not among size classes within a species (*p* > 0.05). CANO mortality was not significantly different among locations (*p* > 0.05). CHICH, Chichagof Island; GBNP, Glacier Bay National Park; KUP, Kupreanof Island; and POW, Prince of Wales Island. Note that for KUP PISI, proportional mortality is pulled from only three trees across all sampled plots, so it is not representative of regional PISI mortality

Mortality was documented in each of the most common conifers on the plot network (*C. nootkatensis, P. sitchensis, T. heterophylla, T. mertensiana;* Figure 2) and across all size classes, but, with the exception of *C. nootkatensis,* proportions were in line with or lower than expectations of snag abundances for the region (Deal, Oliver, & Bormann, 1991; Hennon, Hansen, et al., 1990; Hennon & McClellan, 2003). Proportional mortality in mature *P. sitchensis* was high on Kupreanof Island (Figure 2), but this was driven by a lack of trees on the plot network (two dead of three total trees on eight plots). Mortality of less common *P. contorta* occurred only on Chichagof Island (mean = 30% ± 3% for mature and small trees), and no mortality was documented for *T. plicata*.

*Callitropsis nootkatensis* mortality did occur across all tree size classes (mature, small, and treelet; Figure 2), and estimated time since *C. nootkatensis* mortality ranged from 0 (in healthy stands) to 75 years (Table A1). Mortality was not documented in sapling and seedling size classes, likely due to the short‐lived nature of this fine material. The proportion of dead *C. nootkatensis* averaged 74% (median = 75%, range = 4%–100%) across the extent of decline, while background mortality in healthy stands north of the decline averaged 16% (median = 12%, range = 0.01%–43%). Mortality of common co‐occurring conifers was significantly lower than that of *C. nootkatensis* (Kruskal–Wallis chi‐squared = 160.45, *df* = 3, *p* < 0.001). Canopy mortality was also significantly different among co‐occurring species (Kruskal–Wallis chi‐squared = 33.79, *df* = 3, *p* < 0.001); however, within‐species mortality was not significantly different between size classes (NS within species, Kruskal–Wallis chi‐squared (species pooled) = 2.77, *df* = 2, *p* > 0.05) or locations (NS within species, Kruskal–Wallis chi‐squared (species pooled) = 6.81, *df* = 3, *p* > 0.05).

**Figure 3:**
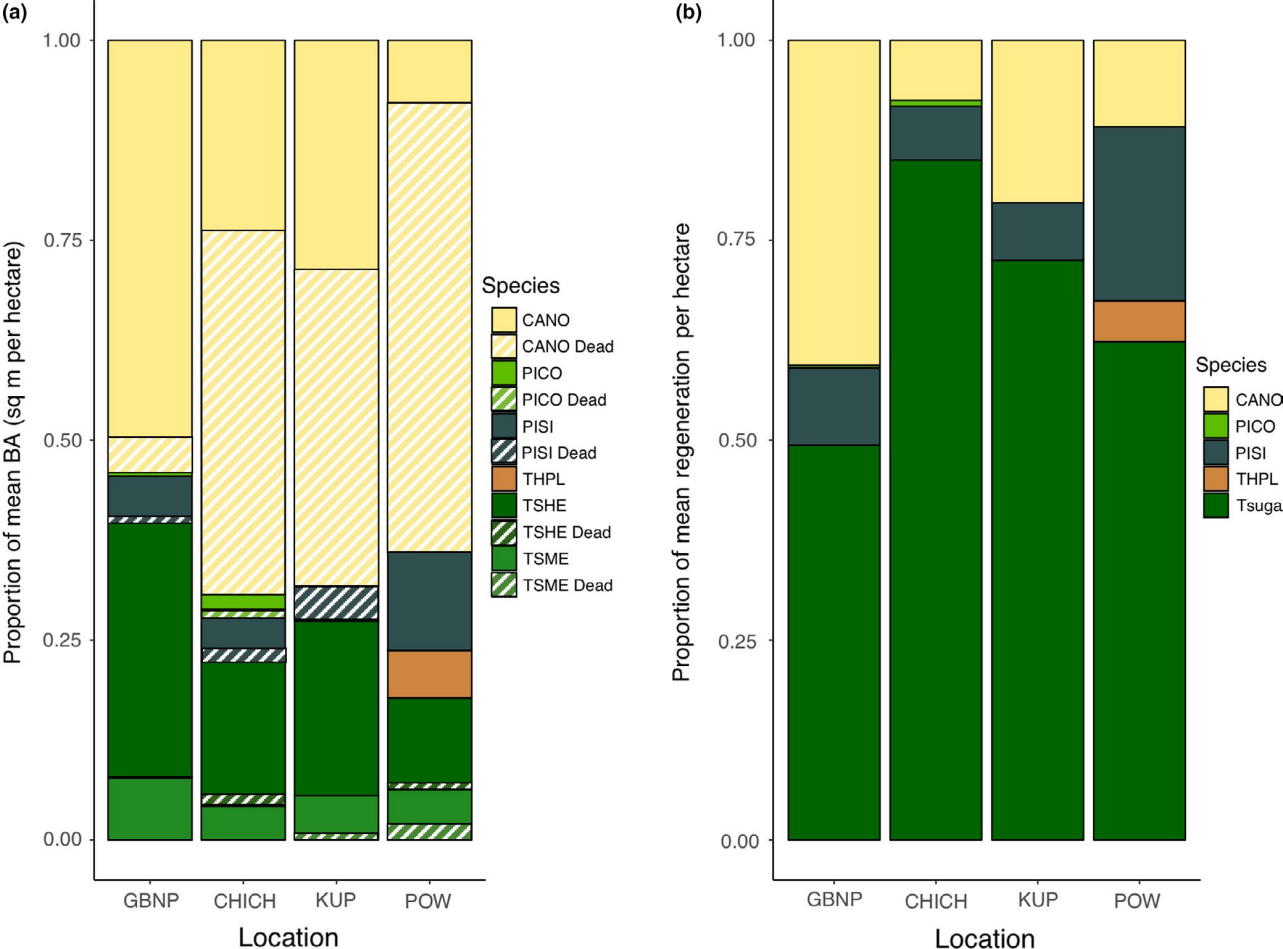
(a) Proportion of average canopy (mature trees, small trees, treelets, and saplings) live and dead basal area per hectare by species and location across southeast *Callitropsis nootkatensis* plot network. (b) Proportion of average regeneration densities (germinants and seedlings per hectare) by species and location across southeast *C. nootkatensis* plot network. CANO, *Callitropsis nootkatensis*; CHICH, Chichagof Island; GBNP, Glacier Bay National Park; KUP, Kupreanof Island; PICO, *Pinus contorta*; PISI, *Picea sitchensis*; POW, Prince of Wales Island; THPL, *Thuja plicata*; *Tsuga*, hemlock species. Community composition was significantly different among locations (*p* < 0.001) for the canopy and regenerating communities and across the severity of *C. nootkatensis* mortality (*p* < 0.05) for the regenerating community

Species abundances were significantly different within both the canopy and regenerating communities. Live tree abundances varied by species (Kruskal–Wallis chi‐squared = 43.17, *df* = 5, *p* < 0.001), but significance was driven by variation in live *C. nootkatensis* among sampling locations (Kruskal–Wallis chi‐squared = 9.83, *df* = 4, *p* < 0.05). Live, mature *C. nootkatensis* continued to dominate the canopy in Glacier Bay National Park (mean dead = 8%), an area currently north of the region of decline, but was a minor component (>50% dead across all plots, mean = 75%) at the southern end of decline on Prince of Wales Island (Figure 3a). Canopy community composition (Figure 3a) varied significantly by location (MANOVA *R* ^2^ = 0.13, *df* = 3, *p* < 0.001) and as a function of the severity of *C. nootkatensis* mortality (MANOVA *R* ^2^ = 0.29, *df* = 3, *p* < 0.001). Regenerating community composition (Figure 3b) was highly variable among locations (MANOVA *R* ^2^ = 0.29, *df* = 3, *p* < 0.001) but closely related to canopy condition (Figure 3a) and the severity of *C. nootkatensis* mortality (MANOVA *R* ^2^ = 0.09, *df* = 3, *p* < 0.05). Areas of high severity *C. nootkatensis* mortality had the lowest abundances of *C. nootkatensis* regeneration (Kruskal–Wallis chi‐squared = 23.02, *df* = 3, *p* < 0.001, Figure 3a, b). *Tsuga* species dominated the regenerating community across all locations and were the principal regenerating species in areas of high decline (Figure 3b).

### 3.2 Climate and community drivers of mortality and regeneration

**Figure 4:**
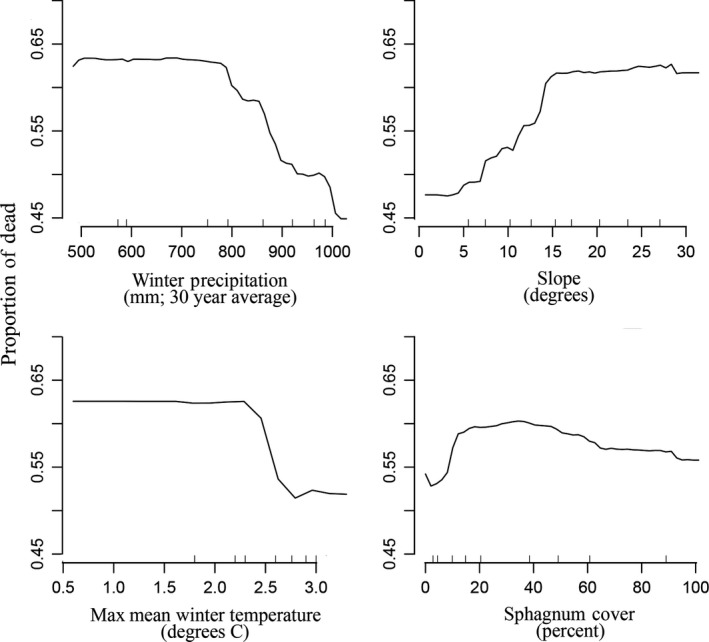
Change in predicted percent of mortality (modeled as proportion of dead *Callitropsis nootkatensis*) on the plot network as driven by the top four most significant uncorrelated variables in the final model. Y‐axis is the modeled mortality percentage as a function of the overall random forest model while varying the top for variables, respectively. For example, mortality generally declines in areas of higher winter precipitation and lower slopes

In the random forests model, higher *C. nootkatensis* mortality was correlated with cool winter temperatures and lower winter precipitation as well as two metrics of soil drainage—moderate *Sphagnum* percentage and higher slopes (mean squared residuals = 0.06, variance explained = 0.40; Figure 4). As a result, the highest proportional mortality on the plot network was found in cooler, relatively drier regions and on higher productivity upland forests.

**Figure 5:**
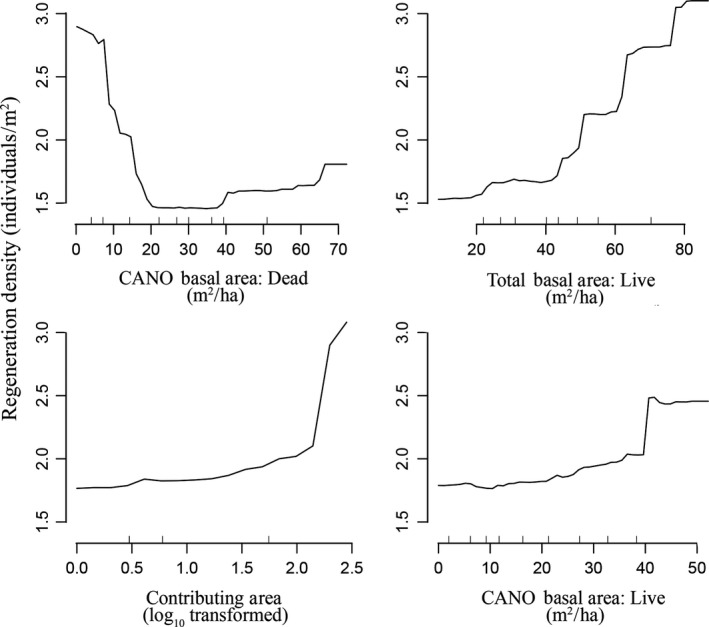
Change in predicted *Callitropsis nootkatensis* regeneration densities on the plot network as driven by the top four most significant uncorrelated variables in the final model. There is a clear relationship between forest health, seed source, and regeneration, with higher densities seen in areas of lower *C. nootkatensis* mortality, higher live *C. nootkatensis* (presumably seed source), and higher overall basal area. Higher densities are also found in wetter landscapes, with a higher contributing area

**Figure 6:**
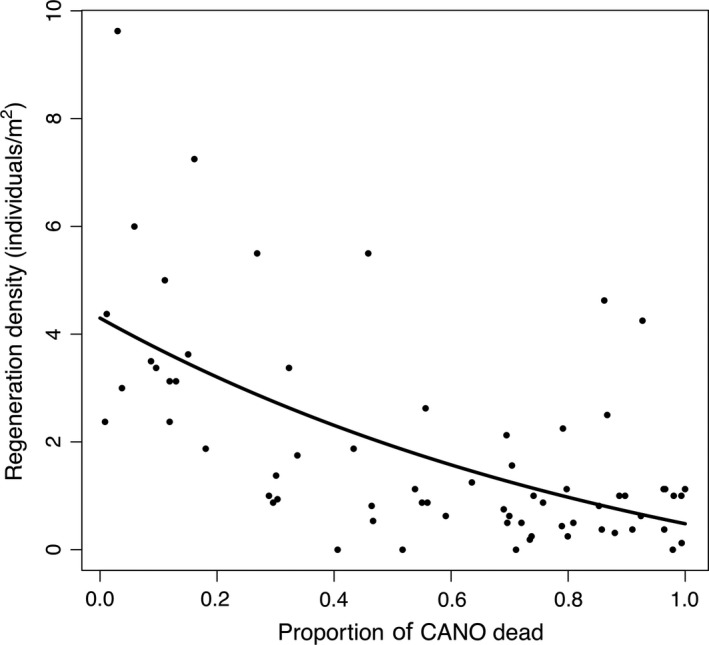
*Callitropsis nootkatensis* (CANO) regeneration densities (individuals per square meter) per plot (black, filled circles) as a function of the proportion of dead *C.nootkatensis* in the canopy. Regeneration densities decline with increasing severity of canopy mortality (*p* < 0.001)

*Callitropsis nootkatensis* regeneration abundances were strongly correlated with stand condition and canopy composition (variance explained = 0.45; mean squared residuals = 2.02). The top four variables in the model were basal area of dead and live *C. nootkatensis,* total live stand basal area (all species), and contributing area (Figure 5). *Callitropsis nootkatensis* basal area was the most important variable in explaining regeneration abundance, with higher regeneration in areas with a greater proportion of live *C. nootkatensis* in the canopy and a lower proportion of dead *C. nootkatensis* (Figure 5). This was corroborated by the direct comparison between canopy mortality and regeneration; regeneration (log‐transformed) was negatively correlated with increasing mortality (*p* < 0.001, *r* ^2^ = 0.38, *F* = 40.64 on 1,65 *df*; Figure 6).

### 3.3 Response and stability in regenerating community composition

**Figure 7:**
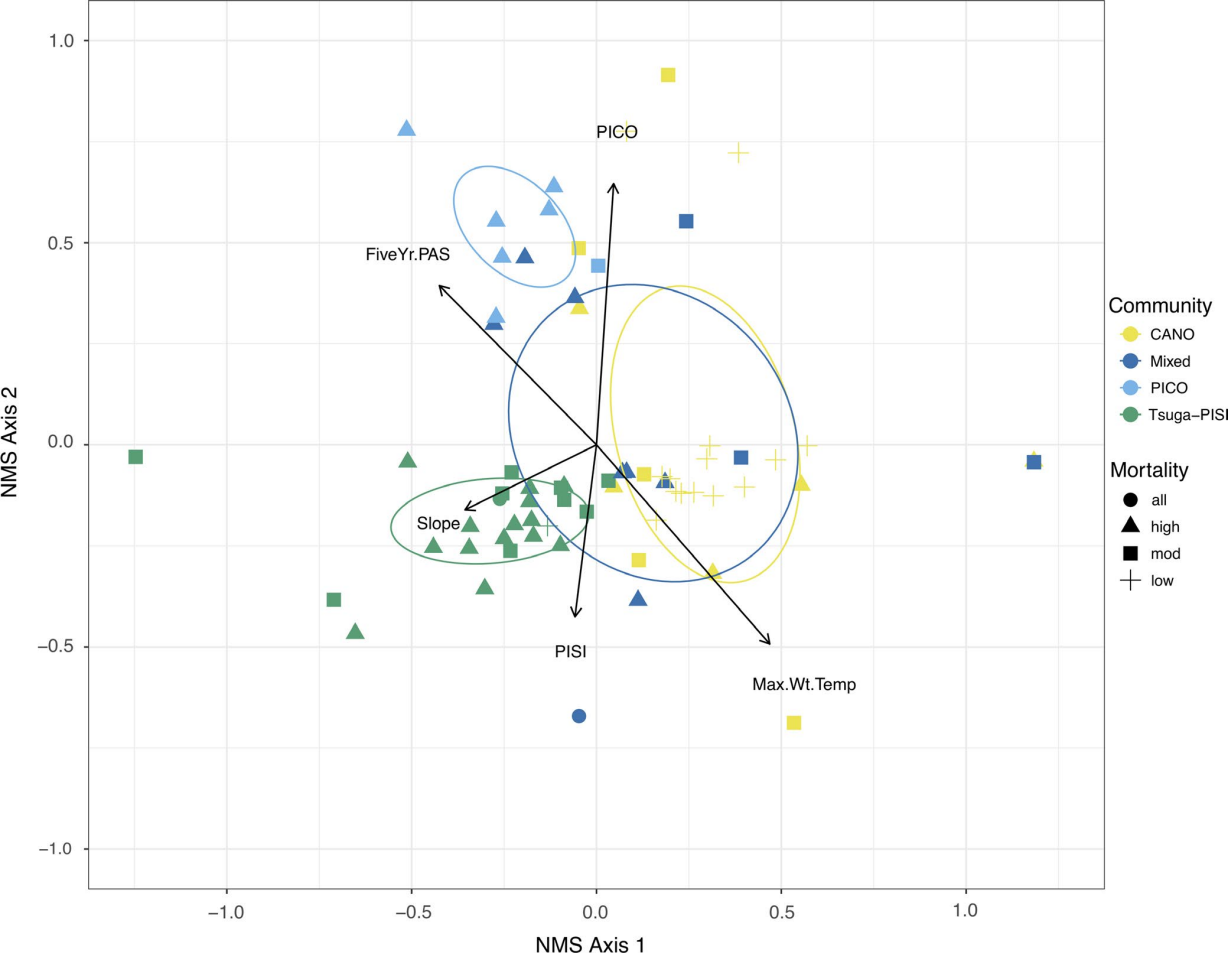
Nonmetric multidimensional scaling (NMS) of the regenerating community by classification of the severity of *Callitropsis nootkatensis* canopy mortality (shapes) and community type (colors), with each point representing individual sample plots. Mortality severity classes: low = 1%–25%, moderate = 25.1%–69.9%, high = 70%–99%, and all = 100%. Ellipses represent the mean scores of each NMS community cluster. Species labels represent the most abundant species in each NMS community cluster. CANO, *Callitropsis nootkatensis*; PICO, *Pinus contorta* spp. *contorta*; PISI, *Picea sitchensis*; THPL, *Thuja plicata*; and Tsuga, *Tsuga heterophylla* and *mertensiana*. Variables and associated vector arrows indicate the direction and magnitude of effects of the canopy community, local stand conditions, and local annual and five‐year climate on regenerating community composition. Arrow length is proportional to the magnitude of correlation. Variables identified as both significant (*p* < 0.05) and minimally correlated include Max.Wt.Temp = maximum winter temperature (°C), Five.Yr.PAS, five‐year average precipitation as snow (mm), average plot slope (°), PICO, *Pinus contorta* spp. *contorta* basal area; and PISI, *Picea sitchensis* basal area

Four regenerating community types were identified in the cluster and indicator species analysis. Community types were largely determined by extent of *C. nootkatensis* decline, with communities assigned to mixed‐conifer (Mixed), *C. nootkatensis* (CANO), *Tsuga‐P. sitchensis* (Tsuga‐PISI), or *P.contorta* (PICO) clusters (Figure 7). The Mixed type was highly variable in location, climate, and drainage condition (no significant indicator species scores (ISS), *p* > 0.05; Figure 7). The CANO type occurred in low slope, lower‐productivity plots in areas of low *C. nootkatensis* mortality (ISS = 70, *p* < 0.01). The Tsuga‐PISI type was found on steeper slope, higher productivity plots and consisted of *Tsuga* (ISS = 81, *p* < 0.01) and *P. sitchensis* (ISS = 72, *p* < 0.05) in areas of moderate to complete *C. nootkatensis* mortality. The PICO type was assigned to low slope, lower‐productivity peatlands with high bryophyte coverage (ISS = 60, *p* < 0.05). Plots of similar condition generally clustered into the same community types; plots with high to complete *C. nootkatensis* mortality were regenerating to Tsuga‐PISI or PICO, with *C. nootkatensis* regeneration dominating in low mortality, lower‐productivity peatlands. Location (Kruskal–Wallis chi‐squared = 19.07, *df* = 3, *p* < 0.001), severity of *C. nootkatensis* mortality (Kruskal–Wallis chi‐squared = 19.94, *df* = 3, *p* < 0.001), and time since mortality (Kruskal–Wallis chi‐squared = 15.78, *df* = 3, *p* < 0.01) were significant predictors of regenerating community types, indicating that climate and both the timing and the extent of decline are primary determinants of community composition stability.

In areas of high *C. nootkatensis* decline, *C. nootkatensis* regeneration was low and replaced by *Tsuga* spp. (Figure 7), and community composition was best explained by the following climatic variables in NMS analysis and vector fitting: maximum winter temperature (*R* ^2^ = 0.30, *p* < 0.001) and five‐year average precipitation as snow (*R* ^2^ = 0.22, *p* < 0.001; Figure 7). Canopy community composition and stand characteristics additionally explained regenerating community composition; *P. contorta* (*R* ^2^ = 0.27, *p* < 0.001) and *P. sitchensis* (*R* ^2^ = 0.12, *p* < 0.05) live basal area plus slope (*R* ^2^ = 0.10, *p* < 0.05) drove community clustering (Figure 7). Variation in community composition was best explained by two axes (final stress = 0.10, nonmetric *R* ^2^ = 0.99, linear *R* ^2^ = 0.96). Axis 1 relates to a gradient of canopy mortality and local hydrologic condition (and associated ecosystem productivity), with *C. nootkatensis* successfully regenerating on low slope peatlands and *Tsuga* species dominating well‐drained upland forests (Figure 7). Axis 2 divides forest ecosystem types by productivity (*P. contorta‐*dominated peatlands from *Tsuga–P. sitchensis* upland forests).

## 4 DISCUSSION

As the climate changes, successful tree regeneration is threatened by novel climate and disturbance conditions and associated shifts in canopy community composition. Our study highlights how species‐specific sensitivity to climate change can lead to restructuring of the forest community following canopy mortality. We demonstrate that reductions in both mature tree and regeneration abundances after single‐species mortality events occur via the same mechanism—death of mature, seed‐producing trees. In the case of *C. nootkatensis*, our work suggests that climate‐induced forest mortality is driving alternate successional pathways in forests where *C. nootkatensis* was once a major component, which is likely to lead to long‐term shifts in community composition and stand dynamics. As suitable habitat conditions shift with ongoing global climate change, both mature trees and regeneration will be increasingly exposed to novel conditions, and identifying the autecological factors contributing to successful regeneration, or lack thereof, is an essential first step toward predicting forest response and resilience to climate change.

### 4.1 Climate impacts on mortality and regeneration in Callitropsis nootkatensis

In the NPCTR, climate change‐induced canopy mortality is restricted to a single species, *C. nootkatensis,* allowing for a focused examination of the effects of climate, seed source, and local conditions on regenerating community response and subsequent stand dynamics. Mature tree mortality across the study area corresponded with a reduction in *C. nootkatensis* regeneration (Figure 6) and a shift to surviving canopy species, predominantly *Tsuga* (Figure 3), a widespread species known to dominate the regenerating community following disturbance (Alaback and Tappeiner II (1991); Deal & Farr, 1994). *Callitropsis nootkatensis* regeneration constituted less than 20% of the regenerating community in decline areas (Figure 3) and appears to be limited by a lack of available seed and vegetative source, although drainage conditions, as determined by slope, and maximum winter temperatures also contribute to structuring regenerating community composition (Figure 7).

Local hydrologic regime and associated ecosystem productivity are key factors in both species’ distributions (Bisbing et al., 2016) and in the extent of *C. nootkatensis* decline (D'Amore & Hennon, 2006). Mortality across this plot network was most extensive on wetter, lower‐productivity peatlands; however, proportional mortality was greatest in productive upland forests. The apparent lack of congruity between these results and previous research, which reports higher mortality on lower slopes (D'Amore & Hennon, 2006), is explained by our use of proportional mortality rather than total. Although mortality is most extreme on saturated peatlands, it is likely that surviving individuals will allow for ongoing perpetuation of the species on lower‐productivity peatlands.

Nonetheless, the loss of *C. nootkatensis* from both the canopy and the regenerating community across the extent of decline in southeast Alaska indicates that canopy trees and regeneration are in sync in their responses to climate change. Cold, low‐snow winters have led to widespread mortality over our study area (Figure 4) and across the species’ range (Buma et al., 2017), and local drainage conditions (i.e., slope, hydrologic regime) amplify or mitigate response of the canopy (Figure 4) and regenerating community (Figures 5 and 7). While this study cannot conclusively determine the mechanism for the lack of regeneration, the close correspondence between the canopy and regenerating communities is striking. These plot‐level findings are consistent with recent modeling work on drivers of canopy mortality that identified winter temperatures and slope as conditions leading to decline at broad scales (Buma et al., 2017). Mortality is predicted to be ongoing in areas above the winter snow threshold, and up to 50% of current suitable habitat is expected to experience climate conditions favorable to decline (Buma et al., 2017). Continued mortality of this conifer is likely to lead to more widespread shifts in community composition (Oakes et al., 2014; data presented here). The close relationship between seed source and composition of the regenerating community suggests that no compensation in the form of increased recruitment will make up for the loss of the *C. nootkatensis* canopy.

### 4.2 Single versus multiple species decline

Mortality events specifically attributed to climate change (Breshears et al., 2005; Van Mantgem & Stephenson, 2007; Williams et al., 2010) do not typically discriminate but, instead, lead to decline or mortality of multiple species in the affected ecosystem. Sierra Nevada mixed‐conifer forests have, for example, seen concurrent declines in shade tolerant *Abies* and shade‐intolerant *Pinus* following years of temperature‐driven drought stress (Van Mantgem & Stephenson, 2007), while extreme droughts in the arid southwest led to mortality in *Pinus edulis* and *Juniperus monosperma* (Mueller et al., 2005). In both cases, no immediate impacts on regenerating community composition were evident, although higher *P. edulis* mortality indicated that long‐term shifts are likely (Mueller et al., 2005). The climate change‐induced *C. nootkatensis* mortality in the NPCTR is unique in its isolated effect on a single species but also in its observable, and now documented, postdecline shift in species dominance.

Regenerating community response in other single‐species mortality events has been highly variable and largely driven by the severity of a biotic disturbance, such as insect attack (Burr & McCullough, 2014), on the canopy community and variability in local establishment conditions (Kayes & Tinker, 2012). In some cases, regeneration proceeds successfully following the loss of canopy species (Diskin, Rocca, Nelson, Aoki, & Romme, 2011; Macek et al., 2017), while, in others, species experience loss of dominance (Pelz & Smith, 2012), increasing the probability of ecosystem type conversions (Burr & McCullough, 2014; Klooster et al., 2014). Self‐replacement of a single species following canopy morality has been documented following extreme drought (Suarez & Lloret, 2018) and bark beetle attack (Diskin et al., 2011), where co‐occurring species established but failed to dominate. These instances of ongoing success, however, occurred in forest types where the impacted species dominated the canopy prior to the disturbance and either did not experience wholesale mortality, as is the case in *Nothofagus* drought mortality (Suarez & Lloret, 2018), or possessed a canopy seed bank, as occurs with *Pinus contorta* ssp. *latifolia* forests (Diskin et al., 2011).

Regeneration declines or failures have also been documented in cases of single‐species mortality (Burr & McCullough, 2014; DeRose & Long, 2007; Klooster et al., 2014; Pelz & Smith, 2012). Laminated root rot in the Northwest has led to similar compositional changes as seen in *C. nootkatensis* forests, where *T. heterophylla* assumes dominance as canopy mortality of *Pseudotsuga menziesii* proceeds (Hansen & Goheen, 2000). Additionally, the 1990s *Dendroctonus rufipennis* attack on *Picea engelmannii* led to *Abies lasiocarpa* and *Populus tremuloides* dominance in the regenerating community, precluding *P. engelmannii* from returning to dominance (DeRose & Long, 2007). As with *C. nootkatensis* response to canopy mortality, the primary limiting factor in all cases is an available seed source.

Regenerating community response to canopy mortality is clearly influenced by a number of factors, such as establishment environment conditions, postdisturbance climate, seed availability, and herbivory—but available seed source is regularly identified as a primary limiting factor, regardless of disturbance type (e.g., fire vs. beetle attack) and magnitude (e.g., single vs. multiple species). Consistent with studies in other forest types and under different climate‐induced disturbances, our findings suggest that seed source (DeRose & Long, 2007; Redmond et al., 2018; Urza & Sibold, 2017) and microsite/establishment environments (Harvey, Donato, & Turner, 2016; Redmond et al., 2018; Urza & Sibold, 2017) are the most important factors in regenerating community response. Species tolerant of postdecline conditions will have a higher likelihood of future dominance, further reducing available seed and vegetative source of the declining species.

Documenting changes in communities following canopy mortality will be crucial to identifying the ecological, and potentially economic, consequences of these losses. Identifying successional trajectories, in particular, will reduce the large uncertainty around the long‐term impacts of climate change‐driven forest mortality events. Forest response to novel disturbance conditions will be hard to predict, and the lessons learned from these cases can help shift our expectations of postdisturbance stand dynamics, particularly in climate‐impacted systems.

### 4.3 Ecosystem resilience and transition to alternate stable states

The consequences of rapid climate change on forest resilience remain uncertain and are likely to be highly variable, based on a particular forest's ecological memory (i.e., information legacies of ecological adaptations to disturbance; Johnstone et al., 2016). For forests to be resilient and resist transitions to alternate states, there must be synchrony between both information legacies (i.e., genetic adaptations to disturbance, like sprouting in avalanche‐prone ecosystems) and material legacies (i.e., the physical legacies, such as seedbanks, that are present after a disturbance event; Johnstone et al., 2016). Historically, *C. nootkatensis’* shallow root system and early response to spring warming were beneficial, allowing for early, rapid growth each growing season (Hennon et al., 2016); however, this once advantageous legacy is now a deleterious adaptation. Mortality in mature trees limits the potential for biological inertia (Young et al., 2019) due to a lack of available seed. Instead, advanced regeneration via vegetative reproduction remains a successful strategy even in areas of severe decline (unpublished data/personal observation). This may help the species maintain a presence on the landscape during periods of mature tree mortality until periods more favorable for sexual reproduction, similar to the “orphaned cohort” example of *Fraxinus* in response to *Agrilus planipennis* invasion (Klooster et al., 2014). The immediate loss of seed‐producing trees and seedlings (Figures 2 and 3), as well as competition from faster growing species, means these forests are lacking the biological inertia needed for resilience and are likely to transition to *T. heterophylla*‐dominated forests even if *C. nootkatensis* maintains some temporary or disjunct presence on the landscape. For species or forest types undergoing similar climate‐induced mortality events, ecologists will need to determine which legacies contribute to ecosystem resilience, or if novel climate or disturbance will remove these legacies that were historically critical.

Ecosystem resilience may also vary as a function of regional climate fluctuations over space and time, known as transitional climate mortality (Buma, 2018), where mortality is highest within a particular range of climatic conditions but decreases above or below this range. Currently, the mid‐range of the *C. nootkatensis’* distribution is experiencing mortality, but, if emissions scenarios continue toward worst‐case trajectories, it is possible that the mortality “donut‐hole” will be relatively short‐lived (Buma, 2018). Short, intense climate fluctuations may therefore be less deleterious to ecosystem resilience if there are deep enough ecological legacies to sustain species over time.

## 5 CONCLUSIONS AND NEXT STEPS

Widespread mortality of tree species due to changing climate is a major concern in forests worldwide, but the potential for resilience is rarely assessed during an ongoing mortality event, a gap we have attempted to fill here. Species’ responses to climate change‐induced mortality will vary widely based on species‐specific traits, sensitivity to climatic extremes, biotic stressors, and abiotic conditions of the establishment environment, thus requiring autecological studies on factors limiting versus promoting success. In *C. nootkatensis* forests, there is no increase in *C. nootkatensis* regeneration abundances to offset canopy mortality. As a result, this forest type is not resilient to mortality associated with ongoing snow loss, and a type change appears to be underway. This example of climate change‐driven mortality in a single species highlights how species‐specific sensitivity can lead to shifts in community composition and stand dynamics following canopy mortality via the same mechanism—death of mature seed trees.

Few strategies or solutions exist for forests vulnerable to ecosystem type conversion due to mature tree mortality and associated loss of seed source, and numerous knowledge gaps remain. In the case of *C. nootkatensis,* common garden studies using seed sources from across the species’ range could allow for identification of genotypes with fine‐root frost tolerance. Planting gardens across decline severity gradients would also allow for targeted research on regeneration response to concurrent canopy mortality and associated establishment conditions. Given the potential for a wave of transitional mortality across the *C. nootkatensis* range (Buma, 2018), however, survival of local versus foreign seed source is hard to predict. Long‐term monitoring in our plot network will allow us to track forest response to the predicted transitional mortality phenomenon and provide demographic information on the impacts of climate on different size classes as well as on the growth and survival rates of regeneration across the decline severity gradient. Demographic studies will also resolve the confounding effects of seed availability, canopy mortality, and establishment conditions on regeneration. Simultaneously investigating climate‐induced tree mortality and subsequent postmortality resilience gives a clearer view of the long‐term consequences of climate change on forest health, and this observational study has just scratched the surface of filling the critical knowledge gaps essential to understanding and predicting long‐term forest resilience to climate change.

## CONFLICT OF INTEREST

None declared.

## AUTHORS CONTRIBUTION

A.B., B.B, and S.B. conceived of the study. A.B, B.B., S.B., J.K, and L.E.O. collected the data. B.B. and S.B analyzed the data. S.B. led the writing with contributions from all authors.

## Data Availability

All plot‐level and summary data generated for this study are available through the Dryad Digital Repository (https://doi.org/10.5061/dryad.23420f6).

## APPENDIX 1

**Table A1:** Plot‐level locational and compositional information from across network in southeast Alaska, USA

Plot ID	Location	Year	Latitude	Longitude	Dominant tree species ‐ live BA	Time since CANO death (yrs)	CANO live BA per ha	CANO dead BA per ha	CANO regeneration per m^2^	CANO regeneration per ha
1.1	KUP	2015	56.85047°N	133.53033°W	*C. nootkatensis*	35	38.0	26.0	0.0	0
1.2	KUP	2015	56.85076°N	133.53135°W	*T. heterophylla*	35	8.0	30.0	0.4	4,375
1.3	KUP	2015	56.85082°N	133.53009°W	*C. nootkatensis*	35	39.0	37.0	0.9	9,375
1.4	KUP	2015	56.85062°N	133.53244°W	*C. nootkatensis*	35	24.0	1.0	0.5	5,333
2.1	KUP	2015	56.85022°N	133.52821°W	*C. nootkatensis*	35	18.0	26.0	0.6	6,250
2.2	KUP	2015	56.84827°N	133.52614°W	*C. nootkatensis – T. heterophylla*	35	10.0	58.0	0.8	8,125
2.3	KUP	2015	56.84755°N	133.52513°W	*T. heterophylla*	35	3.0	22.0	0.3	3,125
2.4	KUP	2015	56.84936°N	133.52615°W	*C. nootkatensis*	35	15.0	13.0	0.8	8,125
3.1	POW	2016	56.21524°N	133.26588°W	*C. nootkatensis*	10	12.2	33.8	0.2	1875
3.2	POW	2016	56.21558°N	133.26656°W	*P. sitchensis*	10	0.3	47.0	1.0	10,000
3.3	POW	2017	56.21528°N	133.26707°W	*P. sitchensis*	10	17.4	41.4	1.6	15,625
3.4	POW	2017	56.21476°N	133.26645°W	*T. mertensiana*	10	17.3	14.6	5.5	55,000
4.1	POW	2016	56.22578°N	133.27412°W	*T. heterophylla*	10	2.6	31.9	0.6	6,250
4.2	POW	2016	56.22504°N	133.27389°W	*P. sitchensis*	10	6.1	39.7	2.5	25,000
4.3	POW	2017	56.22577°N	133.27365°W	*T. heterophylla*	10	15.2	37.4	<0.01	1
4.4	POW	2017	56.22473°N	133.27292°W	*C. nootkatensis*	10	25.1	26.9	<0.01	1
5.3	POW	2017	55.91775°N	132.85709°W	*T. heterophylla*	35	5.5	70.0	4.3	42,500
6.3	POW	2017	56.03667°N	133.23947°W	*T. heterophylla*	35	9.2	22.2	NA	NA
GB_010_066	GBNP	2011	58.22538°N	136.63204°W	*C. nootkatensis*	0	31.7	4.7	3.2	31,250
GB_011_071	GBNP	2011	58.24562°N	136.61262°W	*C. nootkatensis*	0	24.5	0.2	2.4	23,750
GB_012_064	GBNP	2011	58.21917°N	136.63780°W	*T. heterophylla*	0	29.5	5.2	3.6	36,250
GB_014_068	GBNP	2011	58.23110°N	136.62305°W	*C. nootkatensis*	0	45.4	5.7	5.0	50,000
GB_02_024	GBNP	2011	58.29816°N	136.67510°W	*C. nootkatensis*	0	52.2	0.6	4.4	43,750
GB_03_019	GBNP	2011	58.28641°N	136.68011°W	*C. nootkatensis*	0	41.7	1.3	9.6	96,250
GB_04_042	GBNP	2011	58.27781°N	136.71230°W	*C. nootkatensis*	0	41.8	2.6	6.0	60,000
GB_07_016	GBNP	2011	58.28207°N	136.67820°W	*C. nootkatensis*	0	28.1	3.8	3.1	31,250
GB_08_048	GBNP	2011	58.26283°N	136.71370°W	*C. nootkatensis*	0	36.0	3.8	3.4	33,750
GB_09_067	GBNP	2011	58.22911°N	136.62730°W	*C. nootkatensis*	0	44.6	4.3	3.5	35,000
H_02_062	CHICH	2011	57.55681°N	136.01001°W	*C. nootkatensis*	0	35.9	27.5	1.9	18,750
H_03_071	CHICH	2011	57.57203°N	136.04004°W	*C. nootkatensis*	0	35.9	6.9	7.3	72,500
H_04_091	CHICH	2011	57.62267°N	136.12257°W	*C. nootkatensis*	0	35.4	18.1	1.8	17,500
H_06_068	CHICH	2011	57.56611°N	136.02991°W	*C. nootkatensis*	0	37.8	1.5	3.0	30,000
H_08_124	CHICH	2011	57.57882°N	135.96251°W	*C. nootkatensis*	0	33.0	14.2	1.4	13,750
H_09_082	CHICH	2011	57.63800°N	136.07839°W	*T. heterophylla*	0	22.5	8.3	5.5	55,000
H_10_126	CHICH	2011	57.56995°N	135.97246°W	*C. nootkatensis*	0	38.8	5.2	2.4	23,750
H_13_088	CHICH	2011	57.62485°N	136.10913°W	*C. nootkatensis*	0	22.7	9.2	1.0	10,000
H_15_084	CHICH	2011	57.64685°N	136.11008°W	*C. nootkatensis*	0	30.3	12.7	0.9	8,750
H_16_129	CHICH	2011	57.56802°N	135.98041°W	*C. nootkatensis*	0	27.2	6.0	1.9	18,750
M_01_029	CHICH	2011	57.50626°N	135.90751°W	*T. heterophylla*	35	0.6	31.4	1.0	10,000
M_02_057	CHICH	2011	57.54192°N	135.98833°W	*T. heterophylla*	35	3.9	39.2	0.4	3,750
M_03_044	CHICH	2011	57.52819°N	135.96468°W	*T. heterophylla*	35	0.8	35.9	0.0	0
M_04_035	CHICH	2011	57.5157°N	135.91531°W	*T. heterophylla*	35	7.1	22.3	0.9	8,750
M_05_029	CHICH	2011	57.5045°N	135.90228°W	*C. nootkatensis*	35	9.4	26.9	1.0	10,000
M_06_029	CHICH	2011	57.50793°N	135.90987°W	*C. nootkatensis*	35	7.9	22.3	0.3	2,500
M_07_026	CHICH	2011	57.48985°N	135.88183°W	*C. nootkatensis*	35	11.1	28.5	0.5	5,000
M_08_031	CHICH	2011	57.51351°N	135.91613°W	*P. contorta* ssp. *contorta*	35	3.2	28.1	1.0	10,000
M_09_057	CHICH	2011	57.53963°N	135.98675°W	*C. nootkatensis*	35	9.6	22.0	0.5	5,000
M_10_051	CHICH	2011	57.53694°N	135.97525°W	*C. nootkatensis*	35	29.1	37.0	0.9	8,750
O_01_006	CHICH	2011	57.46517°N	135.82757°W	*T. mertensiana*	75	0.2	37.2	0.1	1,250
O_02_010	CHICH	2011	57.46275°N	135.83273°W	*C. nootkatensis*	75	16.3	36.3	0.8	7,500
O_03_022	CHICH	2011	57.4867°N	135.86483°W	*T. heterophylla*	75	6.5	51.4	1.0	10,000
O_04_005	CHICH	2011	57.45955°N	135.81151°W	*T. heterophylla*	75	6.9	43.0	4.6	46,250
O_05_028	CHICH	2011	57.49871°N	135.89103°W	*T. heterophylla*	75	1.3	32.8	1.1	11,250
O_06_019	CHICH	2011	57.48018°N	135.85594°W	*T. heterophylla*	75	11.4	14.3	2.6	26,250
O_08_011	CHICH	2011	57.46494°N	135.83600°W	*T. mertensiana*	75	1.1	29.9	0.4	3,750
O_09_010	CHICH	2011	57.46065°N	135.82668°W	*T. heterophylla*	75	21.2	25.9	0.9	8,750
O_10_015	CHICH	2011	57.47464°N	135.83629°W	*T. heterophylla*	75	2.5	72.2	1.1	11,250
O_14_002	CHICH	2011	57.45436°N	135.81272°W	*T. mertensiana*	75	8.5	36.1	0.5	5,000
R_01_133	CHICH	2011	57.55577°N	136.00360°W	*T. heterophylla*	10	13.2	30.1	2.1	21,250
R_02_055	CHICH	2011	57.53862°N	135.98150°W	*C. nootkatensis*	10	21.4	37.3	1.3	12,500
R_03_037	CHICH	2011	57.52466°N	135.93290°W	*C. nootkatensis*	10	15.3	58.2	2.3	22,500
R_04_059	CHICH	2011	57.54938°N	135.98393°W	*C. nootkatensis*	10	27.6	13.2	3.4	33,750
R_06_047	CHICH	2011	57.53040°N	135.96152°W	*C. nootkatensis*	10	9.6	58.0	0.4	3,750
R_07_058	CHICH	2011	57.54386°N	135.99047°W	*C. nootkatensis*	10	10.8	42.9	0.3	2,500
R_08_101	CHICH	2011	57.54978°N	135.95184°W	*C. nootkatensis*	10	20.5	23.9	1.1	11,250
R_09_133	CHICH	2011	57.55844°N	135.99534°W	*T. heterophylla*	10	0.0	62.0	1.1	11,250
R_10_041	CHICH	2011	57.52584°N	135.95378°W	*T. heterophylla*	10	12.9	50.7	1.1	11,250
R_11_059	CHICH	2011	57.54442°N	135.99834°W	*C. nootkatensis*	10	16.9	39.4	0.6	6,250

Abbreviations: BA, basal area (m^2^/ha); CANO, *Callitropsis nootkatensis*; CHICH, Chichagof Island; GBNP, Glacier Bay National Park; KUP, Kupreanof Island; and POW, Prince of Wales Island.
